# Supplementary material for: Dose-Dependent Transcriptional Response to Ionizing Radiation Is Orchestrated with DNA Repair within the Nuclear Space
Source: Int J Mol Sci. 2024 Jan 12;25(2):970. doi: 10.3390/ijms25020970 (PMC10815587; doi:10.3390/ijms25020970)
Supplement: Supplementary file 1 [file ijms-25-00970-s001.zip › Figure S1.pdf]

Figure S2

	PPIB	HCRTR	PRSS35	TRIM29
0h	0.60	0.57	0.46	0.24
1Gy_6h	0.59	0.58	0.50	0.59
1Gy_24h	0.62	0.52	0.47	0.23
6Gy_6h	0.59	0.64	0.48	0.70
6Gy_24h	0.62	0.59	0.45	0.16
