# Supplementary figures and images for: Dose-Dependent Transcriptional Response to Ionizing Radiation Is Orchestrated with DNA Repair within the Nuclear Space
Source: Int J Mol Sci. 2024 Jan 12;25(2):970. doi: 10.3390/ijms25020970 (PMC10815587; doi:10.3390/ijms25020970)

# Figure S1

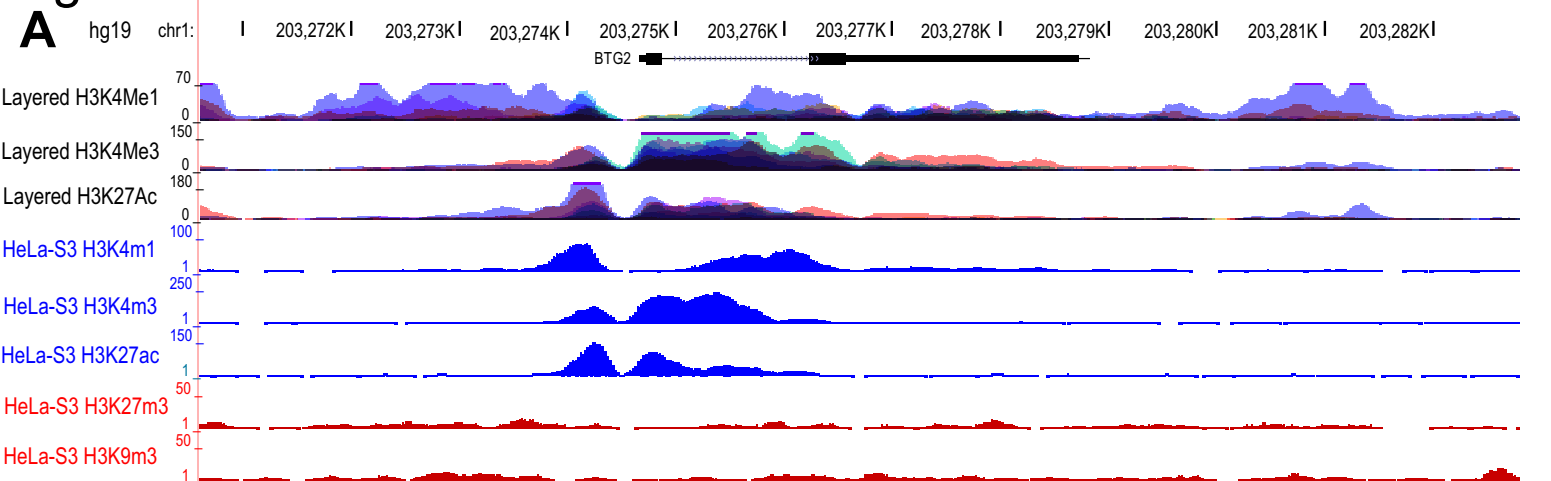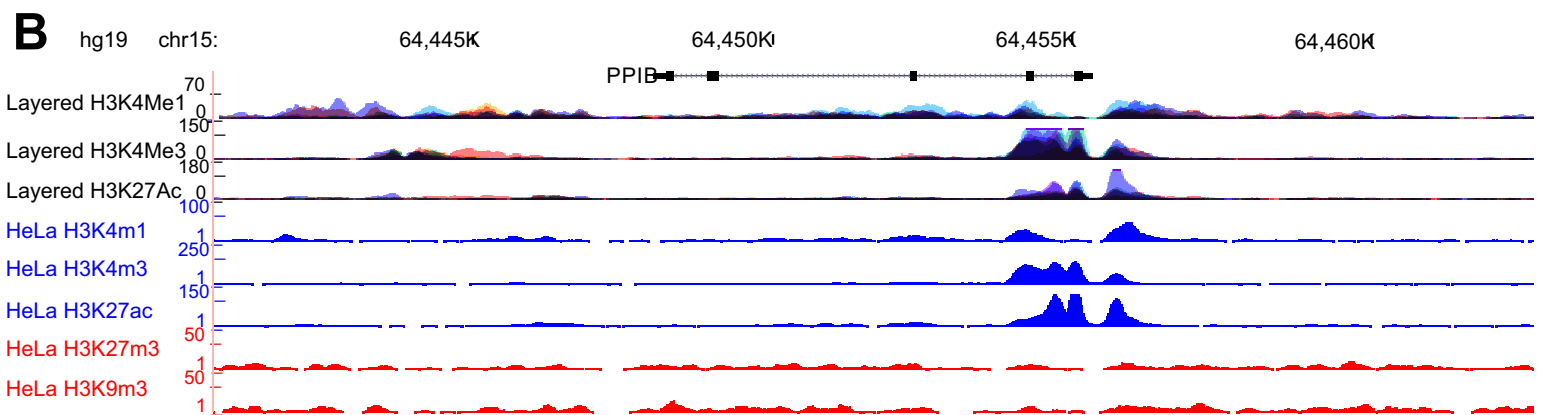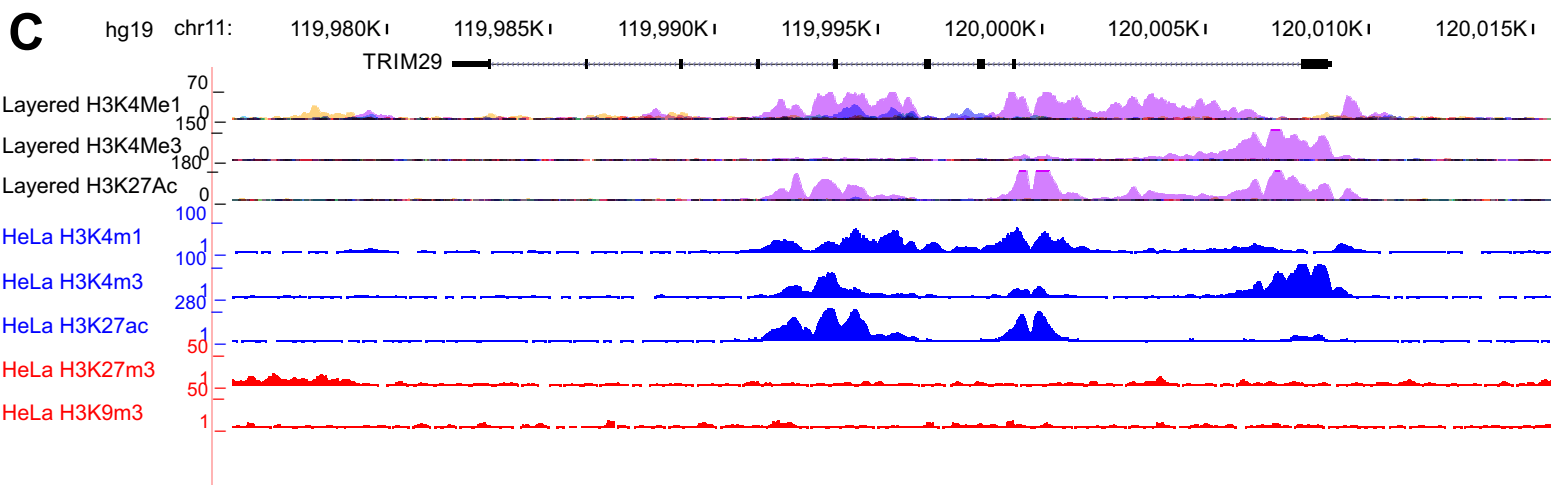

Supplement: Supplementary file 1 [file ijms-25-00970-s001.zip › Figure S2A-C.pdf]

# Figure S1

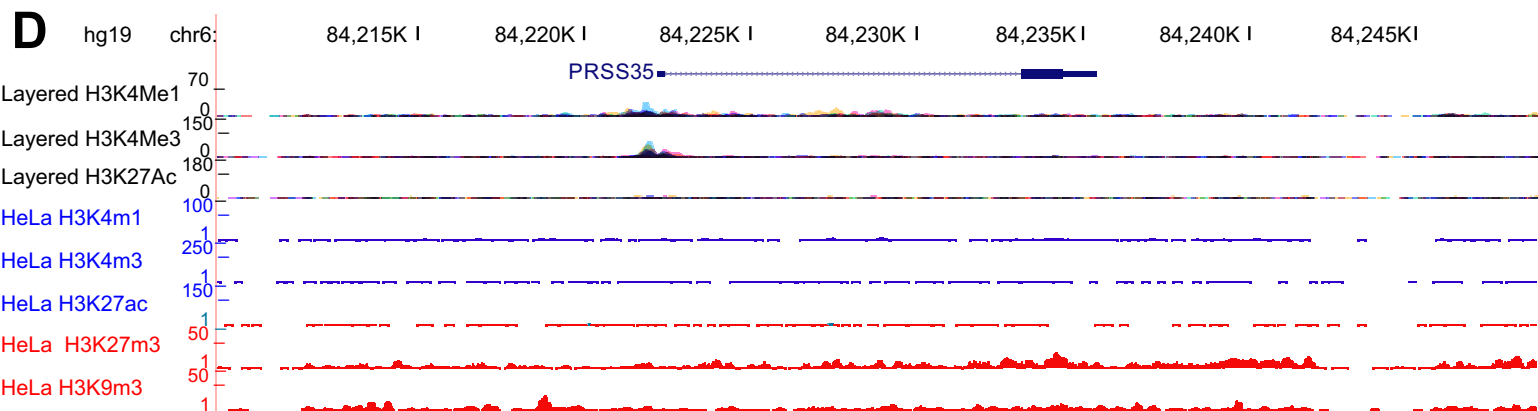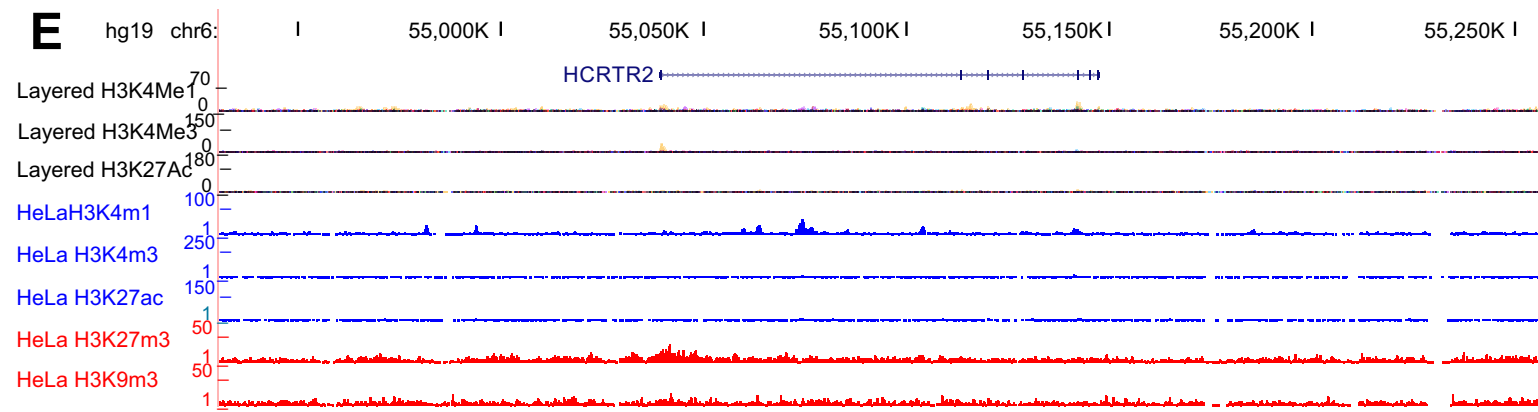

Supplement: Supplementary file 1 [file ijms-25-00970-s001.zip › Figure S2D,E.pdf]
